# Use of a search summary table to improve systematic review search methods, results, and efficiency

**DOI:** 10.5195/jmla.2021.809

**Published:** 2021-01-01

**Authors:** Alison C. Bethel, Morwenna Rogers, Rebecca Abbott

**Affiliations:** 1 a.bethel@exeter.ac.uk, Information Specialist, Evidence Synthesis Team, University of Exeter Medical School, Exeter, United Kingdom; 2 morwenna.rogers@exeter.ac.uk, Evidence Synthesis Team, National Institute for Health Research Applied Research Collaboration South West Peninsula, University of Exeter Medical School, Exeter, United Kingdom; 3 r.a.abbott@exeter.ac.uk, Evidence Synthesis Team, National Institute for Health Research Applied Research Collaboration South West Peninsula,, University of Exeter Medical School, Exeter, United Kingdom

## Abstract

**Background:**

Systematic reviews are comprehensive, robust, inclusive, transparent, and reproducible when bringing together the evidence to answer a research question. Various guidelines provide recommendations on the expertise required to conduct a systematic review, where and how to search for literature, and what should be reported in the published review. However, the finer details of the search results are not typically reported to allow the search methods or search efficiency to be evaluated.

**Case Presentation:**

This case study presents a search summary table, containing the details of which databases were searched, which supplementary search methods were used, and where the included articles were found. It was developed and published alongside a recent systematic review. This simple format can be used in future systematic reviews to improve search results reporting.

**Conclusions:**

Publishing a search summary table in all systematic reviews would add to the growing evidence base about information retrieval, which would help in determining which databases to search for which type of review (in terms of either topic or scope), what supplementary search methods are most effective, what type of literature is being included, and where it is found. It would also provide evidence for future searching and search methods research.

## BACKGROUND

Systematic reviews are designed to be comprehensive, robust, inclusive, transparent, and reproducible when bringing together the evidence to answer a research question. Depending on the field and topic, they may be large and time consuming with many included studies, or they can contain no relevant studies at all, finding that the area urgently requires primary research [1]. The timescales to publication can vary widely [2], and some systematic reviews are regularly updated, particularly if there is new relevant evidence in the field being researched [3]. However, research consistently shows that search strategies are not recorded well enough to allow them to be reproduced [4–6].

Systematic review guidelines recommend that the systematic review team include expertise in systematic review methods, including information retrieval [7–10]. Information retrieval is a core competency for librarians and information specialists who are involved in systematic reviews [11], and having a librarian or information specialist as part of the team is associated with significantly higher-quality search strategies [12]. The role of a librarian or information specialist in a systematic review can vary, ranging from the more limited role of checking searches written by others in the team to taking on many, if not all, aspects of search development and information retrieval [7, 9, 13].

Once a protocol is in place, one of the first tasks undertaken by the librarian or information specialist is to create search strategies for the predefined bibliographic databases listed in the protocol. Ideally, this task is then followed up by supplementary searching, such as forward and backward citation searching or table of contents searching in journals that are relevant to the topic [14]. Searching for grey literature is also recognized as an important part of a comprehensive search strategy for systematic reviews, and previous literature describes which resources are best suited to finding it and the contribution it can make to a systematic review [15–18]. Finally, depending on how long the systematic review takes to publication, update searches may also be part of the process [3].

Guidelines and guidance for conducting systematic reviews are available from various organizations, including Cochrane, the Campbell Collaboration, the Centre for Reviews and Dissemination, and the Joanna Briggs Institute. These guidelines all include some detail about how the searching should be undertaken, but there is no clear consensus about how many or which databases should be searched. Similarly, tools for assessing the quality of systematic reviews vary somewhat in their recommendations of what reviewers should look for in the search methods. Table 1 highlights a few of the different guidelines and checklists for undertaking, reporting, and appraising the searching component of systematic reviews, organized by the tool or author or organization.

**Table 1:** Recommendations for systematic review searching from guidelines and checklists

Organization/Tool	Guidance
Cochrane [19]	Chapter 6 details the search process for Cochrane systematic reviews (SRs): “it is recommended that for all Cochrane reviews CENTRAL and MEDLINE should be searched, as a minimum, together with EMBASE if it is available.”
Campbell Collaboration [8]	Method guide 1 details searching for studies: for database searches, “a search of one database alone is typically not considered adequate.”
Joanna Briggs Institute [10]	“There is inadequate evidence to suggest a particular number of databases, or even to specify if any particular databases should be included.”
Centre for Reviews and Dissemination [7]	“Due to the diversity of questions addressed by systematic reviews, there can be no agreed standard for what constitutes an acceptable search in terms of the number of databases searched.”
Preferred Reporting Items for Systematic Reviews and Meta-Analyses (PRISMA) checklist [20]	“Describe all information sources (e.g., databases with dates of coverage, contact with study authors to identify additional studies) in the search and date last searched,” and “present full electronic search strategy for at least one database, including any limits used, such that it could be repeated.”
A MeaSurement Tool to Assess systematic Reviews (AMSTAR) appraisal tool [21]	Section 4 of the AMSTAR checklist is relevant to the search: it asks whether review authors used a comprehensive literature search strategy and performed the following steps: “Searched at least 2 databases (relevant to research question) Provided key word and/or search strategy Justified publication restrictions (e.g., language) Searched the reference lists/bibliographies of included studies Searched trial/study registries Included/consulted content experts in the field Where relevant, searched for grey literature Conducted search within 24 months of completion of the review.”
Critical Appraisal Skills Programme (CASP) appraisal tool [22]	“Section 3 Do you think all the important, relevant studies were included? HINT: Look for which bibliographic databases were used follow up from reference lists personal contact with experts unpublished as well as published studies non-English language studies.”

As well as understanding where and how to search for information, it is important to understand how well the search strategies perform. Cooper et al. suggest there are six summative metrics of search effectiveness: sensitivity, specificity, precision, accuracy, number needed to read (NNR), and yield [23]. However, while there are suggested standards for reporting search methods and strategies [24–26], there currently are no requirements to report on these effectiveness metrics. Some research has been published on search effectiveness, but this research seems to be restricted to systematic reviews of certain conditions [27–32] or from specific organizations [33–35]. By reporting search effectiveness as well as search methods in more detail, evidence about information retrieval would accumulate, which could then inform guidelines about how many and which databases to search and which supplementary search methods to use for particular topics or types of evidence synthesis.

The aim of this study was to develop a search summary table (SST) that could report on search methods as well as search effectiveness. The authors demonstrate what an SST could look like and how it can be used. In the suggested SST, the only metric not covered by Cooper et al. [23] is “specificity,” because this requires a known number of references (e.g., when developing a search filter).

## CASE PRESENTATION

The SST was tested by the Evidence Synthesis Team at the University of Exeter in a systematic review, “‘They've Walked the Walk': A Systematic Review of Quantitative and Qualitative Evidence for Parent-to-Parent Support for Parents of Babies in the Neonatal Unit” by Hunt et al. [36]. The database search strategies used for the review are provided in supplemental Appendix A, and a blank SST template is provided in supplemental Appendix B.

### Completion of a search summary table (SST)

The SST was completed in two stages. In stage one, all the references that were downloaded or exported from every electronic database, including all duplicates, were recorded and saved in an EndNote library. Every record included a code for the database name where the record was found. As per traditional systematic review methods, the number of records screened at both the title-and-abstract stage and full-text stage were recorded as well as the final number of included references and which supplementary search methods were undertaken. Stage two involved rerunning the searches in those databases where most included references had been found in order to discover whether references that were not found during the original search were in the database and, if they were, whether they were retrieved by the search.

The SST presents the search information used to inform the PRISMA flow diagram, the search methods, and additional information gathered by the librarian or information specialist in their search log. Completion of stage one took approximately forty minutes and completion of stage two approximately one hour. Using this format to present the information allows calculation of various search effectiveness metrics.

Table 2 shows the key features of the SST. The first five metrics (numbered 1 to 5) are summative metrics of effective searching suggested by Cooper et al. [23]. Three additional metrics (numbered 6 to 8) provide further useful search-related information for the librarian or information specialist.

**Table 2:** Metrics used in the search summary table (SST)

Metric	Definition and use in the SST
1. Sensitivity/Recall	Number of relevant references identified by the database search relative to the total number of relevant references found by all search methods. Reported for each database search as well as overall considering the total number of articles screened.
2. Precision	Number of relevant references identified by the database search relative to the total number of references found by all search methods, reported for each database search as well as overall considering the total number of articles screened.
3. Number needed to read (NNR)	Number of references a researcher must screen/read to identify a relevant reference. Equivalent to 1/overall precision. Reported overall and further split into 2 metrics: (1) number needed to screen (NNS) during title and abstract screening to identify 1 reference to undergo full-text screening, and (2) number needed to read during full-text screening (NNR FT) to include 1 reference in the systematic review.
4. Yield	Number of references retrieved by the database search.
5. Format	Reference type (e.g., journal article, doctoral [PhD] thesis). Reported for each reference to show the types of references found in each database.
6. Number of included references	Number of references included in the systematic review.
7. Number of unique references	Number of included references retrieved by a database search that were not retrieved by any other database search.
8. Number of references screened	Number of references screened from each database, which depends on the order in which de-duplication was performed.

Sensitivity/recall and precision calculations are given in the SST for each database searched and overall, using the total number of references that have been found from database searching, the number of included (i.e., relevant) references from database searching, and the total number of included (i.e., relevant) references from all search methods. Reporting these metrics in this manner shows the effectiveness of search strategies for each individual database as well as database searching as a whole.

NNR usually indicates the number of references needed to screen at the title and abstract stage to find one included reference. However, the value of splitting this metric into two additional metrics can be seen: (1) number needed to screen (NNS), which is the number of references that needed to be screened during title and abstract screening to identify one reference to undergo full-text screening; and (2) number needed to read at full text (NNR FT), which is the number of references that needed to be read during full-text screening to include one reference in the systematic review. Reporting these three metrics separately increases the transparency of the searching and selection process.

Table 3 shows the SST for Hunt et al.'s systematic review [36].

**Table 3:** Completed SST for Hunt et al.'s systematic review, ‘“They've Walked the Walk': A Systematic Review of Quantitative and Qualitative Evidence for Parent-To-Parent Support for Parents of Babies in Neonatal Care” [36]

		Databases searched (date run: June 2017, date rerun: January 2019)											Supplementary searches				
Included reference	Format	ASSIA	BNI	CINAHL	Cochrane (2 databases)	EMBASE	HMIC	MED-LINE	PQDT	Psyc-INFO	SPP	WoS* (3 databases)	fcs	bcs	hs	wss	org
Ardal 2011 (qL)	jnl					x		x		n							
Livermore 1980 (qL)	jnl					n		n		x							
Macdonell 2013 (qL)	jnl			x				x		x							
Merewood 2006 (qT)	jnl				x	x		x		n							
Minde 1980 (qT)	jnl				x	x		x		n							
Morris 2008 (qL)	ths			x		n		n		x							
Niela-Vilen 2016 (qT)	jnl			x		n		x		x		x					
Oza-Frank 2014 (qT)	jnl					z		z		n			x	x			
Preyde 2001 (qL)	jnl					n		n		n				x			
Preyde 2003 (qT)	jnl			x	x	x		x		x		x					
Preyde 2007 (qT)	jnl	x				x	x	x		x	x	x					
Roman 1988 (qL)	ths					n		n		x							
Roman 1995 (qT)	jnl			x		x		x		x		x					
Rossman 2011 (qL)	jnl					z		z		n			x	x			
Rossman 2012 (qL)	jnl	x				y		x		x							
No. included refs		2	0	5	3	6	1	9	0	9	1	4	2	3			
No. unique refs		0	0	0	0	0	0	0	0	2	0	0	0	1			
Yield		563	90	771	530	1,887	50	1,803	109	1,182	14	602					
No. refs screened		451	82	613	122	495	34	1,797	86	692	0	221					
Sensitivity		13.33	0	33.33	20	40	6.67	60	0	60	6.67	26.67					
Precision		0.36	0	0.65	0.57	0.32	2	0.5	0	0.76	7.14	0.66					
No. of database searched				14													
Sum of yields				7,431						Overall sensitivity		80					
No. of refs that underwent title and abstract screening				4,593						Overall precision		0.26					
No. of refs that underwent full-text screening				118						NNR		383					
No. of included refs from database searching				12						NNR FT		10					
Total no. included refs				15						NNS		39					

* Social Sciences Citation Index, Conference Proceedings Citation Index–Science, and Conference Proceedings Citation Index-Social Sciences and Humanities.

Codes: x=found from the search; y=in database, found when search strategy rerun; n=not in the database; z=in the database, not found using the search strategy; qL=qualitative; qT=quantitative.

Format codes: jnl=journal article; ths=PhD thesis.

Supplementary search codes: fcs=forward citation search; bcs=backward citation search; hs=hand search; wss=website search; org=from contacting organizations.

Databases listed: ASSIA=Applied Social Sciences Index and Abstracts; BNI=British Nursing Index; HMIC=Health Management Information Consortium; PQDT=ProQuest Dissertations and Theses; SPP=Social Policy and Practice; WoS=Web of Science.

### Contextual consideration of the findings

Key findings can be surmised from the metrics reported in the SST for this example systematic review, which involved a search for both qualitative and quantitative evidence.

#### Grey literature.

Two doctoral (PhD) theses were included in the systematic review, and both were found by searching in PsycINFO. One was also found by searching CINAHL. This was surprising, because grey literature searching is often seen as separate from the database searching process, yet these theses were found by searching bibliographic databases as opposed to Proquest Dissertations and Theses Global.

#### Search strategy comprehensiveness.

Three included references were found from citation searching, two of which were in both EMBASE and MEDLINE but were not retrieved by the database search strategies. If the search strategies had included the free-text search term “council*” (supplemental Appendix A), then these references would have been retrieved. This was an extremely valuable learning point for the information specialist in the team and reaffirmed the purpose of supplementary searching.

#### Unique references.

The only database to retrieve unique references (n=2) was PsycINFO, demonstrating the high degree of duplication among bibliographic databases.

#### Supplementary searching.

Hand searching, website searching, and organization searching was carried out but found no additional relevant references. Although forward citation searching found two additional relevant references, both of these (and a third additional relevant reference) were also found by backward citation searching. The time spent on these methods of supplementary searching was not recorded but might be useful in the future.

#### Qualitative references.

The CINAHL search retrieved only two of the eight qualitative references and did not retrieve any unique qualitative references. This was surprising, because previous research showed that this database was a good source of qualitative studies [37].

#### Quantitative references.

All the quantitative references were found from searching MEDLINE and citation searching.

#### Number needed to read.

Reporting the overall NNR as well as splitting this metric into two metrics—NNS and NNR FT—allowed more accurate and transparent reporting of the screening stages. Concerning the NNS, for every thirty-nine references that underwent title and abstract screening, one underwent full-text screening. Concerning the NNR, for every ten references read in full-text screening, one was included in the systematic review.

Often MEDLINE and EMBASE are suggested as the basic minimum for searching on health care topics [7, 19]; however, in this case, neither database provided unique records, although MEDLINE had higher sensitivity and precision than EMBASE. For this particular systematic review, searching MEDLINE and PsycINFO along with backward citation searching would have found all of the included references. If this had been done, then the maximum number of references to screen would have been 2,835 (total number of references downloaded), a reduction of over 1,600.

The optimal number of databases that need to be searched varies depending on the review question. However, it is commonly agreed that searching only one database is not sufficient, and supplementary searching in some form is also needed. In this case, backwards citation searching found additional studies.

## DISCUSSION

An SST can report data related to database and search performance and effectiveness in terms of sensitivity, precision, recall, NNR, yield, and number of unique records. Furthermore, additional information gathered during supplementary searching (e.g., citation searching and hand-searching) indicates the effectiveness of search strategies for individual databases and which methods of supplementary searching were most useful. This information could allow librarians and information specialists to be more selective when choosing databases and supplementary search methods. Publishing an SST as part of a systematic review would help to develop and make more explicit, rather than tacit, the model of the literature search process as described by Cooper et al. [38].

Future systematic reviews on a similar condition or population could use a related SST that is already available, either in-house or one that has been published, to enable a more evidence-based approach to database and search methods selection. For rapid reviews [39] and scoping reviews [40], in which searching might not be as exhaustive, this information could provide evidence about where to focus the search. SSTs would be particularly valuable in updating a systematic review [3]; if, for example, two databases that are always searched consistently do not contain any of the included studies, then perhaps they need not be searched in the future.

An SST only provides evidence for one particular systematic review; teams using them for future systematic reviews might not be fully confident that the same results would be produced for their specific questions. However, if all systematic reviews completed and published an SST as standard, then there would be more evidence available for making evidence-based decisions on which databases to search, which supplementary search methods would be most valuable, and which search strategies and terms would find the most relevant references for specific questions. Broad generalizations on searching cannot be made until more SSTs are available, but they can still be a valuable learning tool for all those involved in searching for systematic reviews, as their creation requires reflection on what was done and why, which can be carried on into the next systematic review. SSTs can also provide evidence for other purposes of searching, such as update searches [3] or scoping and preliminary searches [40].

SSTs can be useful to librarians and information specialists in several ways. First, for individuals who are new to the topic area or to systematic reviews, they provide a valuable source of evidence on which to base database and search method choices and recommendations. Second, they provide evidence about which databases are essential for undertaking specific systematic reviews, which could be useful for groups or individuals in negotiating database access with their institutions. Third, SSTs could help librarians and information specialists audit their database selections and search strategies, as they would show whether a database contains a reference and whether it would be captured by their search strategy. Fourth, a librarian or information specialist's knowledge would be built up more quickly, because completing an SST would help them reflect on their search strategies, search methods, and database selection.

Another area in which SSTs could be useful is in search methods and information retrieval research. If SSTs are published as part of a systematic review, then the searching becomes more transparent, replicable, and open, which is a fundamental component of good quality systematic reviews. Librarians and information specialists could use the data provided in SSTs to perform more thorough analyses on where studies are likely to be found and which databases suit particular topics. Trends might be observed, such as country-specific biases in database selection and use, and knowledge about specific databases could be shared in an easy format.

One specific area for monitoring is grey literature. By reviewing and analyzing SSTs, librarians and information specialists would be able to determine the extent to which grey literature publications are included in systematic reviews and how they are found, which would help to focus search time and energy. Future research following from this project may include finding a simple way to retrospectively evaluate search strategies, which could help improve future search strategy or search methods development or aid in the creation of a repository where all SSTs could be shared and accessed.

Cooper et al.'s systematic review identified fifty studies of the effectiveness of literature searching, which was a representative sample of the available literature [23]. SSTs would add to this literature and help move forward the discussion about what constitutes an effective search for a systematic review.

The SST is a simple way to collate the search information generated from a systematic review. Creating and reporting an SST as part of a systematic review would add to the knowledgebase on database selection and supplementary search methods and provide evidence for future searching and search methods research.

## SUPPLEMENTAL FILES

**Appendix A:** MEDLINE searches

**Appendix B:** Blank search summary table (SST) template
