# Supplementary material for: Use of a search summary table to improve systematic review search methods, results, and efficiency
Source: J Med Libr Assoc. 2021 Jan 1;109(1):97–106. doi: 10.5195/jmla.2021.809 (PMC7772975; doi:10.5195/jmla.2021.809)
Supplement: Supplementary file 1 — Appendix A: MEDLINE searches [file jmla-109-1-97-s01.pdf]

JMLA Journal of the Medical Library Association

SC
Supplemental content to
J Med Libr Assoc. Jan;109(1):dx.doi.org/10.5195/jmla.2021.809
www.jmla.mlanet.org
© Bethel, Rogers, Abbott 2021

Use of a search summary table to improve systematic review search methods, results, and efficiency

Alison C. Bethel; Morwenna Rogers; Rebecca Abbott

APPENDIX A
MEDLINE searches

Original MEDLINE search

1. peer.tw.
2. parent*.tw.
3. befriend*.tw.
4. face to face.tw.
5. one to one.tw.
6. mum$1.tw.
7. mother*.tw.
8. dad$1.tw.
9. father*.tw.
10. caregiver*.tw.
11. group*.tw.
12. network*.tw.
13. program*.tw.
14. meeting*.tw.
15. match.tw.
16. friendship.tw.
17. neonat*.tw.
18. nicu.tw.
19. scbu.tw.
20. Special care baby unit*.tw.
21. nnu.tw.
22. adjusted age.tw.
23. corrected age.tw.
24. exp Intensive Care, Neonatal/
25. exp Intensive Care Units, Neonatal/
26. premature.tw.
27. preterm.tw.
28. pre term.tw.
29. weeker.tw.
30. low birth weight.tw.
31. lbw.tw.

32. vlbw.tw.
33. baby.tw.
34. babies.tw.
35. infan*.tw.
36. newborn*.tw.
37. graduate*.tw.
38. survivor*.tw.
39. *Self-Help Groups/
40. 1 or 2 or 3 or 4 or 5 or 6 or 7 or 8 or 9 or 10
41. 11 or 12 or 13 or 14 or 15 or 16
42. ((peer or parent* or befriend* or face to face or one to one or mum$1 or mother* or dad$1 or father* or caregiver*) adj2 (group* or network* or program* or meeting* or match or friendship)).tw.
43. support*.tw.
44. mentor*.tw.
45. (support* adj2 (group* or network* or program* or meeting* or match or friendship)).tw.
46. 41 or 43
47. (mentor* adj2 (group* or network* or program* or meeting* or match or friendship or support*)).tw.
48. ((peer or parent* or befriend* or face to face or one to one or mum$1 or mother* or dad$1 or father* or caregiver*) adj2 support*).tw.
49. ((peer or parent* or befriend* or face to face or one to one or mum$1 or mother* or dad$1 or father* or caregiver*) adj2 mentor*).tw.
50. 39 or 42 or 45 or 47 or 48 or 49
51. 26 or 27 or 28 or 29 or 30 or 31 or 32
52. 33 or 34 or 35 or 36 or 37 or 38
53. ((premature or preterm or pre term or weeker or low birth weight or lbw or vlbw) adj2 (baby or babies or infan* or newborn* or graduate* or survivor*)).tw.
54. 17 or 18 or 19 or 20 or 21 or 22 or 23 or 24 or 25 or 53
55. 50 and 54

Suggested alternative MEDLINE search after completed of the search summary table (SST)

1. peer.tw.
2. parent*.tw.
3. befriend*.tw.
4. face to face.tw.
5. one to one.tw.
6. mum$1.tw.
7. mother*.tw.
8. dad$1.tw.
9. father*.tw.
10. caregiver*.tw.
11. group*.tw.

SC
Supplemental content to
J Med Libr Assoc. Jan;109(1):dx.doi.org/10.5195/jmla.2021.809
www.jmla.mlanet.org
© Bethel, Rogers, Abbott 2021

12. network*.tw.
13. program*.tw.
14. meeting*.tw.
15. match.tw.
16. friendship.tw.
17. **council*.tw.**
18. neonat*.tw.
19. nicu.tw.
20. scbu.tw.
21. Special care baby unit*.tw.
22. nnu.tw.
23. adjusted age.tw.
24. corrected age.tw.
25. exp Intensive Care, Neonatal/
26. exp Intensive Care Units, Neonatal/
27. premature.tw.
28. preterm.tw.
29. pre term.tw.
30. weeker.tw.
31. low birth weight.tw.
32. lbw.tw.
33. vlbw.tw.
34. baby.tw.
35. babies.tw.
36. infan*.tw.
37. newborn*.tw.
38. graduate*.tw.
39. survivor*.tw.
40. *Self-Help Groups/
41. 1 or 2 or 3 or 4 or 5 or 6 or 7 or 8 or 9 or 10
42. 11 or 12 or 13 or 14 or 15 or 16 or 17
43. **((peer or parent* or befriend* or face to face or one to one or mum$1 or mother* or dad$1 or father* or caregiver*) adj2 (group* or network* or program* or meeting* or match or friendship or council*)).tw.**
44. support*.tw.
45. mentor*.tw.
46. **(support* adj2 (group* or network* or program* or meeting* or match or friendship or council*)).tw.**
47. 42 or 44
48. **(mentor* adj2 (group* or network* or program* or meeting* or match or friendship or council* or support*)).tw.**
49. ((peer or parent* or befriend* or face to face or one to one or mum$1 or mother* or dad$1 or father* or caregiver*) adj2 support*).tw.

JMLA Journal of the Medical Library Association

SC
Supplemental content to
J Med Libr Assoc. Jan;109(1):dx.doi.org/10.5195/jmla.2021.809
www.jmla.mlanet.org
© Bethel, Rogers, Abbott 2021

50. ((peer or parent* or befriend* or face to face or one to one or mum$1 or mother* or dad$1 or father* or caregiver*) adj2 mentor*).tw.
51. 40 or 43 or 46 or 48 or 49 or 50
52. 27 or 28 or 29 or 30 or 31 or 32 or 33
53. 34 or 35 or 36 or 37 or 38 or 39
54. ((premature or preterm or pre term or weeker or low birth weight or lbw or vlbw) adj2 (baby or babies or infan* or newborn* or graduate* or survivor*)).tw.
55. 18 or 19 or 20 or 21 or 22 or 23 or 24 or 25 or 26 or 54
56. **51 and 55**
